# Outpatient heart failure specialist care following acute heart failure hospitalisation improves long-term outcomes

**DOI:** 10.1136/openhrt-2025-003432

**Published:** 2025-09-29

**Authors:** Jonathan Raby, Hesham Aggour, Manju George, Hannah Glatzel, Benjamin Fidock, Molly Winter, Karmen Quek, Saul Federman, Josephine Chaplin, Mari Kononen, Nicola Bowers, Andrew Money-Kyrle, Norman Qureshi, Rodney De Palma, Soroosh Firoozan, Punit Ramrakha, Piers Clifford, Mayooran Shanmuganathan

**Affiliations:** 1 Cardiology, Wycombe Hospital, Buckinghamshire Healthcare NHS Trust, High Wycombe, Buckinghamshire, UK; 2 Division of Cardiovascular Medcine, Radcliffe Department of Medicine, University of Oxford, Oxford, Oxfordshire, UK

**Keywords:** HEART FAILURE, Echocardiography, Epidemiology

## Abstract

**Background:**

Hospitalisation with acute heart failure (AHF) carries a high risk of death, and those surviving to discharge remain at high risk of death or rehospitalisation with AHF. The impact of outpatient heart failure (HF) specialist care after discharge from AHF hospitalisation on longer-term outcomes is unclear in contemporary UK practice.

**Methods:**

A retrospective analysis of a cohort of 2104 patients admitted to hospital due to AHF between 2014–2022 in a single UK county was performed. Patient characteristics, left ventricular ejection fraction (EF) (LVEF) category (HF with reduced EF (LVEF ≤40%; HFrEF), HF with mildy reduced EF (LVEF 41%–49%; HFmrEF) and HF with preserved EF (LVEF ≥50%; HFpEF)) and survival free from a composite end point of AHF rehospitalisation or death are described. Cox regression survival analysis was performed to explore the impact of baseline patient characteristics and HF specialist care on long-term outcomes.

**Results:**

The median age of the cohort was 83 years. HFrEF was present in 36%, HFmrEF in 9% and HFpEF in 55%. 13% died during index AHF hospitalisation. Median follow-up for those surviving to discharge was 618 (IQR 264–1275) days. 21% were rehospitalised due to AHF, and 63% died during follow-up. On adjusted survival analysis of 1511 patients with echocardiogram data available, HF specialist care after discharge from hospital was independently associated with a significant reduction in the composite end point across all LVEF categories (HFrEF: HR 0.577, 95% CI 0.429 to 0.775, p<0.001; HFmrEF: HR 0.485, 0.281–0.834, p=0.009; HFpEF HR 0.762, 0.623–0.931, p=0.008).

**Conclusions:**

HF specialist care after discharge for patients hospitalised with AHF is associated with a significant reduction in the long-term risk of rehospitalisation and all-cause death. This association was present across the three LVEF categories (HFrEF, HFmrEF and HFpEF) and was independent of age and important comorbidities.

**WHAT IS ALREADY KNOWN ON THIS TOPIC**

- Hospitalisation due to acute heart failure (AHF) is associated with high morbidity and mortality. Despite advances in heart failure (HF) management, in-hospital and postdischarge outcomes for AHF patients remain poor. Input from a specialist multidisciplinary HF team is recommended at all stages of the HF trajectory to reduce the risk of death and further episodes of AHF requiring rehospitalisation.

**WHAT THIS STUDY ADDS**

- This study provides real-world data on the benefits of outpatient heart failure specialist care following hospitalisation for AHF by evaluating post-discharge outcomes (death or rehospitalisation with AHF) beyond the typical 30-day or 1-year follow-up periods. We further demonstrate that improved outcomes are seen with HF team input in all left ventricular ejection fraction (LVEF) subgroups, even after adjusting for important comorbidities.

**HOW THIS STUDY MIGHT AFFECT RESEARCH, PRACTICE OR POLICY**

- This study highlights the importance of systematic referral for ongoing HF specialist care when a patient is discharged from hospital following a AHF hospitalisation. Our data support the expansion of outpatient multidisciplinary HF specialist care to all HF patients, irrespective of LVEF (About half of such services in the UK are currently only available to patients with reduced LVEF), age and comorbidity status. In drawing attention to the very high rates of adverse outcomes that follow hospitalisation due to AHF, our study should also prompt researchers and policymakers to consider measures that would improve early detection of HF and prevent a first hospitalisation due to AHF.

## Background

 Acute heart failure (AHF) accounts for over 63 500 hospital admissions per year in the United Kingdom (UK).^1^ Despite significant advances in therapies, the last 20 years have produced only modest improvements in heart failure (HF) outcomes.^2^ In the UK, patients hospitalised due to AHF have an approximately 11% chance of dying as an inpatient, and 30% of those that survive die within the following year.^1^ In-hospital and 1-year postdischarge outcomes can be improved by the involvement of a specialist HF team during^3^ and after^4 5^ an AHF hospitalisation. However, the longer term (beyond 1 year) impact of HF specialist care is less well described. Furthermore, while previous analyses have substratified HF patients by left ventricular ejection fraction (LVEF),^6 7^ the correlation of other important phenotypic characteristics with long-term clinical outcomes is less clear. In this study, we offer a detailed analysis of AHF patients admitted to hospitals in a single county in the UK with a population of around 550 000^8^ over an 8-year period. We report detailed cardiovascular phenotypes for these patients based on echocardiography and correlate these and other clinical characteristics with longer term outcomes (death due to any cause and rehospitalisation due to AHF). We explore the long-term prognostic impact of HF specialist care during and after hospitalisation due to AHF.

## Methods

Patients admitted to hospitals in Buckinghamshire, UK with AHF between March 2014 and April 2022 were identified retrospectively as a service evaluation audit approved by the institutional clinical governance department (Cardio/CA/2023-24/01). Demographics, comorbidity, length of stay, discharge medication regimen and follow-up arrangement data were obtained soon after each patient’s discharge. Cardiac phenotype (biventricular systolic and diastolic function, chamber dimensions, presence of valvular disease) was determined from the echocardiogram performed closest to date of index admission. Follow-up attendance, medication regimen at last clinical contact and readmission and mortality data as of 19 December 2022 were determined from electronic records. Where cause of death information was available, cardiovascular death was defined as being due to HF, myocardial infarction, arrhythmia or stroke as recorded in part 1 of the death certificate (ie, identified as the immediate cause of death). AHF hospitalisation events had to fulfil two criteria to be included: (1) primary admission diagnosis of AHF in clinical coding and (2) confirmation by a HF specialist nurse that AHF was the primary reason for the admission based on a retrospective review of available clinical information. Post-discharge HF specialist care in our institution comprises scheduled outpatient face to face or telemedicine appointments with a team of HF specialist nurses working under the direct supervision of a consultant cardiologist with a subspecialty interest in HF.

To explore outcomes across LVEF categories, the cohort was substratified for survival analysis: (1) LVEF ≤40% (HF with reduced ejection fraction, ‘HFrEF’), (2) LVEF 41%–49% (HF with mildly reduced EF, ‘HFmrEF’), (3) LVEF ≥50%. Given the importance of distinguishing patients with primary valvular HF and LVEF ≥50% from HF with preserved EF (HFpEF) as a distinct pathobiological entity,^9^ we performed survival analysis only on patients with the latter. The HFA-PEFF score was calculated for this cohort.^10^ The requirement for echocardiogram data to guide LVEF substratification and exclusion of patients with severe primary valvular heart disease and normal LVEF necessitated restriction of survival analysis to 1511 patients.

Guideline-directed medical therapy (GDMT) for HFrEF was determined from the European Society of Cardiology guidelines,^11 12^ with doses expressed as percentage of the target guideline dose. HFrEF GDMT prescription compliance was assessed against ‘three pillars’ of (1) renin angiotensin system (RAS) inhibitor or angiotensin receptor neprilysin inhibitor (ARNI), (2) beta blocker and (3) mineralocorticoid receptor antagonist (MRA), given that sodium glucose cotransporter-2 inhibitor therapy did not appear in HF guidelines until 2021 for HFrEF and 2023 for HFpEF.

Data analysis was conducted with SPSS (IBM, V.29.0.0.0) and R (V.4.4.1, R Foundation for Statistical Computing). Data are summarised as mean±SD if normally distributed, or else as median (quartile 1–quartile 3). Means were compared with independent two-sample t-test and medians with the Mann-Whitney U test. Comparisons of more than 2 means or medians were performed using Analysis of Variance (ANOVA) or appropriate non-parametric equivalent. Proportions were compared with χ^2^ test. Univariable Cox regression analysis using a composite endpoint of rehospitalisation due to AHF or death from any cause was performed for variables of interest (online supplemental table 2). Variables were included in a multivariable model if p<0.1 on univariable analysis. Multicollinearity of variables included in the final model was assessed using variance inflation factor analysis. The proportional hazards’ assumption was assessed using the Schoenfeld residual test; where this assumption was violated (HFrEF and HFmrEF models), an alternative parametric model (accelerated failure time, Weibull distribution) was used to confirm results. Cases with missing data were excluded listwise from survival analyses.

## Results

### HF patient baseline characteristics and cardiovascular phenotype

Between March 2014 and April 2022, 2104 patients underwent an unplanned index hospital admission for AHF. Baseline cohort characteristics are summarised in table 1. Patients with HFrEF were younger (HFrEF age 79 (69–86) years; HFmrEF 81 (73–88) years; LVEF ≥50% 83 (76–88) years; p<0.001) and more likely to be male (68% vs 56% vs 45%; p<0.001) when compared with HFmrEF and patients with LVEF ≥50%. Ischaemic cardiomyopathy was more prevalent in the HFrEF and HFmrEF subgroups (45% vs 44% vs 38%; p=0.010) while hypertension (55% vs 66% vs 65%; p<0.001) and atrial fibrillation (41% vs 48% vs 51%; p<0.001) were less prevalent in HFrEF.

**Table 1:** Baseline demographic, comorbidity, haemodynamic and biochemical status for all patients at time of index heart failure hospitalisation

	Total cohort (n=2104)	LVEF≤40% (n=622)	LVEF 41–49% (n=165)	LVEF≥50% (n=956)	P value
Age, median (IQR) (count of available data)	82.5 (74.0, 88.0) (n=2104)	79.0 (69.0, 86.0)^1,2^ (n=622)	81.0 (73.0, 87.5) (n=165)	83.0 (76.0, 88.0) (n=956)	<0.001*
Male sex, n (%)	1115 (53%) (n=2104)	421 (68%)^1,2^ (n=622)	93 (56%) (n=165)	434 (45%) (n=956)	<0.001†
Caucasian ethnicity, n (%)	1558 (74%) (n=1706)	445 (72%)^1,2^ (n=493)	124 (75%) (n=136)	716 (75%) (n=789)	0.038†
Past medical history, n (%)					
Ischaemic heart disease	831 (40%) (n=1976)	278 (45%)^2^ (n=591)	73 (44%) (n=159)	359 (38%) (n=907)	0.009†
ICD or CRT device therapy	146 (7%) (n=2104)	101 (16%)^1,2^ (n=622)	10 (6%)^3^ (n=165)	21 (2%) (n=956)	<0.001†
Hypertension	1270 (60%) (n=2025)	340 (55%)^2^ (n=597)	109 (66%) (n=161)	626 (65%) (n=935)	<0.001†
Diabetes mellitus	706 (34%) (n=2026)	207 (33%)^2^ (n=599)	54 (33%)^3^ (n=162)	353 (37%) (n=936)	0.015†
Cerebrovascular accident	83 (4%) (n=2025)	22 (4%) (n=597)	9 (5%) (n=161)	43 (4%) (n=935)	0.611†
eGFR<45 mL/min/1.73 m^2^	370 (18%) (n=1420)	111 (18%) (n=418)	27 (16%) (n=117)	173 (18%) (n=662)	0.860
COPD	280 (13%) (n=1998)	66 (11%)^2^ (n=592)	23 (14%) (n=159)	152 (16%) (n=925)	0.023†
Atrial fibrillation	972 (46%) (n=2104)	253 (41%)^2^ (n=622)	80 (48%) (n=165)	487 (51%) (n=956)	<0.001†
Haemodynamics					
Heart rate (bpm), median (IQR)	79 (65–93) (n=1954)	80 (68–97) (n=619)	80 (68–95) (n=156)	80 (69–91) (n=953)	0.113*
Systolic blood pressure (mm Hg), median (IQR)	127 (108–147) (n=1959)	121 (104–141) (n=581)	129 (116–145) (n=159)	134 (116–154) (n=889)	0.092*
Biochemistry					
Haemoglobin (g/L), mean±SD	120±33 (n=2017)	123±20^1^ (n=600)	121±20^3^ (n=162)	117±20 (n=916)	<0.001†
Urea (mmol/L), median (IQR)	9.5 (6.6–15.0) (n=2090)	9.7 (7.0–15.3) (n=615)	9.3 (6.3–13.1) (n=164)	9.6 (6.5–14.5) (n=900)	0.134*
Creatinine (µmol/L), median (IQR)	107 (81–148) (n=2090)	114 (84–154) (n=615)	99 (81–139) (n=164)	106 (81–144) (n=900)	0.678*
eGFR (mL/min), mean±SD	67±32 (n=1420)	69±31 (n=418)	67±27 (n=117)	66±30 (n=662)	0.494†
Serum sodium (mmol/L), median (IQR)	138 (135–141) (n=2047)	138 (135–141) (n=617)	138 (135–141) (n=163)	138 (135–141) (n=927)	0.070*
Serum potassium (mmol/L) median (IQR)	4.2 (3.8–4.6) (n=1993)	4.2 (3.8–4.6) (n=604)	4.0 (3.7–4.5) (n=156)	4.2 (3.8–4.5) (n=902)	0.284*
NT-proBNP (ng/L), median (IQR)	5230 (2475–12615) (n=670)	7940 (4276–18709) (n=212)	6023 (2664–11831) (n=62)	4241 (2010–8617) (n=328)	0.570*

Each superscript number denotes a significant difference between columns on post-hoc pairwise comparison (p<0.05 after applying Bonferroni correction): (1) LVEF ≤40% vs LVEF 41–49%; (2) LVEF ≤40% vs LVEF≥50%; (3) LVEF 41–49% vs LVEF≥50%.

* Independent samples Kruskal-Wallis one-way ANOVA (analysis of variance).

† χ2 test.

‡ NT-proBNP, N-terminal prohormone of brain natriuretic peptide

COPD, chronic obstructive pulmonary disease; CRT, cardiac resynchronisation therapy; eGFR, estimated glomerular filtration rate; ICD, implantable cardioverter-defibrillator; LVEF, left ventricular ejection fraction.

A comprehensive echocardiogram dataset was available for 1743 patients (83%). These data are summarised in online supplemental table 1. 622 patients (36%) had HFrEF, 165 (9%) had HFmrEF, and 956 (55%) had LVEF≥50%; true ‘HFpEF’ without severe valvular disease was present in 881 (92%) of these patients (this subcohort had a median HFA-PEFF score of 6 (IQR 5–6]).

At the time of admission, haemodynamic and biochemical parameters were similar across LVEF subcategories (table 1) except for serum haemoglobin, which was higher in the HFrEF and HFmrEF groups (123±20 g/L vs 121±20 g/L vs 117±20 g/L, p<0.001).

### In hospital outcomes

The median length of inpatient admission due to AHF was 5 days (IQR 2–12). This did not differ significantly across LVEF categories (HFrEF 5 (2–12) days, HFmrEF 4 (2–12) days, LVEF ≥50% 5 (2–12) days; p=0.876). HFrEF patients were more likely to be admitted under specialist cardiology care (65% vs 47% vs 43%, p<0.001). 1294 (61%) of patients received input from the HF team during admission. Patients with HFrEF were more likely to be reviewed by the HF team (75% vs 60% vs 55%, p<0.001). Longer staying patients (>5 days) were no more likely to be reviewed by the HF team (61% vs 61%, p=0.461).

270 patients (13%) died during the index admission with AHF. For patients with echocardiogram data (166, 61%), death rates were similar across LVEF subgroups (9% vs 9% vs 10%; p=0.677). Cause of death was available for 227 (84%) inpatient deaths, of which 197 (87%) were due to cardiovascular causes. Cardiovascular causes accounted for a greater proportion of total deaths in the HFrEF subgroup (92% vs 69% vs 79%; p=0.051).

### GDMT optimisation prior to discharge

Of 565 HFrEF patients who survived to discharge, 53 (9%) were not on any GDMT, 149 (26%) on one drug, 192 (32%) on 2 drugs and 181 (32%) on all three drugs. By individual drug class, 303 (54%) were prescribed an RAS inhibitor, 458 (83%) a beta blocker and 295 (53%) MRA. SGLT2 inhibitors were prescribed to 28 (5%). 509 patients (90%) were discharged on a loop diuretic, with a median dose equivalent to 80 (40–80) mg of furosemide.

Of 150 HFmrEF patients who survived to discharge, 12 (8%) were not on any GDMT, 40 (27%) on one drug, 66 (44%) on two drugs and 32 (21%) on all three drugs. By individual drug class, 64 (43%) were prescribed an RAS inhibitor, 111 (76%) a beta blocker and 54 (37%) MRA. SGLT2 inhibitors were prescribed in three cases (2%). 133 patients (89%) were discharged on a loop diuretic, with a median dose equivalent to 80 (40–80) mg of furosemide.

Of 862 patients with LVEF ≥50% who survived to discharge, 299 (25%) were prescribed an RAS inhibitor, 531 (65%) a beta blocker, 225 (27%) MRA and 5 (1%) SGLT2 inhibitor. 766 patients (89%) were discharged on a loop diuretic, with a median dose equivalent to 80 (40–80) mg of furosemide.

### Post-discharge care

1834 patients survived to discharge following index AHF admission. Postdischarge HF specialist care was arranged for 1131 patients (61%). Patients referred for postdischarge HF specialist care were on average younger (80 (71–85) years vs 84 (75–89) years, p<0.001), more likely to be male (58% vs 46%, p<0.001) and more likely to receive implantable cardioverter-defibrillator or cardiac resynchronisation therapy (10% vs 4%, p<0.001) than those not receiving postdischarge HF team input. The prevalences of other comorbidities were similar between groups (ischaemic heart disease 43% vs 40%, p=0.288; hypertension 62% vs 65%, p=0.297; diabetes mellitus 35% vs 35%, p=0.804, previous cerebrovascular accident 14% vs 20%, p=0.075; atrial fibrillation 45% vs 48%, chronic obstructive pulmonary disease (COPD) 14% vs 16%, p=0.168). Serum creatinine was slightly lower among those referred for HF team care (101 (84–141) µmol/L vs 105 (75–142) 105 (75–142) µmol/L, p=0.047), but there were no significant differences in other baseline biochemical parameters (serum haemoglobin 121±21 g/L vs 118±49 g/L, p=0.480; serum sodium 138 (136–140) mmol/L vs 137 (134–141) mmol/L, p=0.621; serum potassium 4.0 (3.7–4.5) mmol/L vs 4.2 (3.8–4.5) mmol/L, p=0.583; serum NT-proBNP 5468 (2519–12315) ng/L vs 4825 (2445–10747) ng/L, p=0.434).

When stratified by LVEF, HF specialist care was arranged for 79% of HFrEF patients, 77% of HFmrEF and 53% of HFpEF patients. The median time to first outpatient appointment was 43 (20–114) days. This interval was shorter for HFrEF (36 (15–96) days) and HFmrEF (35 (19–88) days) compared with HFpEF (46 (22–132) days; p=0.006). 38% of patients did not attend their first planned outpatient appointment after discharge. Those not attending appointments were on average older (non-attendee median age 80 (69–86) years vs attendee 77 (68–84) years, p=0.021) but were similar in ethnicity (87 vs 89% Caucasian, p=0.639) and sex distributions (60% vs 58% male, p=0.415). Non-attendees were more likely to have a prior diagnosis of ischaemic heart disease (47% non-attendees vs 37% attendees, p=0.003), but other comorbidities were similar between groups (hypertension 64% vs 62%, p=0.573; diabetes mellitus 39% vs 35%, p=0.210; prior cerebrovascular accident 15% vs 11%, p=0.374). The proportions of those from each LVEF subgroup failing to attend appointments were similar (35% HFrEF, 37% HFmrEF, 36% HFpEF).

Final medication regimen data were available for 469 HFrEF patients, at a median time of 465 (183–948) days since discharge from hospital. At this final encounter, 42 patients (9%) were not prescribed any GDMT, 122 (26%) one drug, 146 (31%) two drugs and 159 (34%) all three drugs. 216 (46%) had no change in the number of GDMT medications prescribed between discharge and last encounter, 100 (21%) were deprescribed GDMT and 153 (33%) had the number of prescribed GDMT drugs increased.

HFrEF patients receiving specialist HF care postdischarge (389, 83%) were more likely to be prescribed two (129 (33%) vs 17 (21%), p=0.036) or three (143 (37%) vs 16 (20%), p=0.004) GDMT drugs at the time of last clinical encounter.

### Long-term outcomes

1834 patients survived to discharge from index HF admission, with a median follow-up duration of 618 (264–1275) days. 1511 of these patients had an echocardiogram performed on index admission documenting LVEF.

During follow-up, rehospitalisation due to AHF occurred in 393 cases (21%). Rehospitalisation rates were similar across cohorts: HFrEF 25% (10.9 rehospitalisations per 100 patient years), HFmrEF 21% (8.6 per 100 patient years), HFpEF 23% (9.4 per 100 patient years) (p=0.526). However, the interval to first rehospitalisation was shorter for HFrEF (74 (17–366) days) than HFmrEF (224 (25–579) days) or HFpEF (256 (69–704) days; p<0.001). The median number of AHF readmissions for each patient was 1 (1–2) across all LVEF categories.

1149 patients (63%) died during follow-up (25.3 deaths per 100 patient years), with no significant difference between LVEF subgroups: 58% HFrEF (25.8 deaths per 100 patient years) versus 57% HFmrEF (23 deaths per 100 patient years) versus 59% HFpEF (25.3 deaths per 100 patient years), p=0.768. Information on cause of death was available for 453 patients (39%), of which 208 (46%) were due to cardiovascular causes. Cardiovascular causes accounted for a greater proportion of HFrEF deaths (55% vs 45% HFmrEF vs 39% HFpEF, p=0.015). Median survival from all-cause death post-AHF hospitalisation was 1043 days (95% CI 951 to 1135) by Kaplan-Meier estimate. Survival duration was not significantly different across LVEF categories (p=0.723): HFrEF 980 days (95% CI 846 to 1114), HFmrEF 1031 days (830–1232), HFpEF 1054 days (916–1192).

The composite endpoint (all-cause death or AHF rehospitalisation) occurred in 1251 patients (68%). Median survival free from the composite endpoint was 748 days (95% CI 660 to 836). Event-free survival was numerically shorter for HFrEF (694 days, 542–845) compared with HFmrEF (904 days, 657–1151) and HFpEF (762 days, 646–878), but this was not significantly different (p=0.804).

### Factors affecting long-term outcomes

#### HF with reduced EF

**Figure 1:**
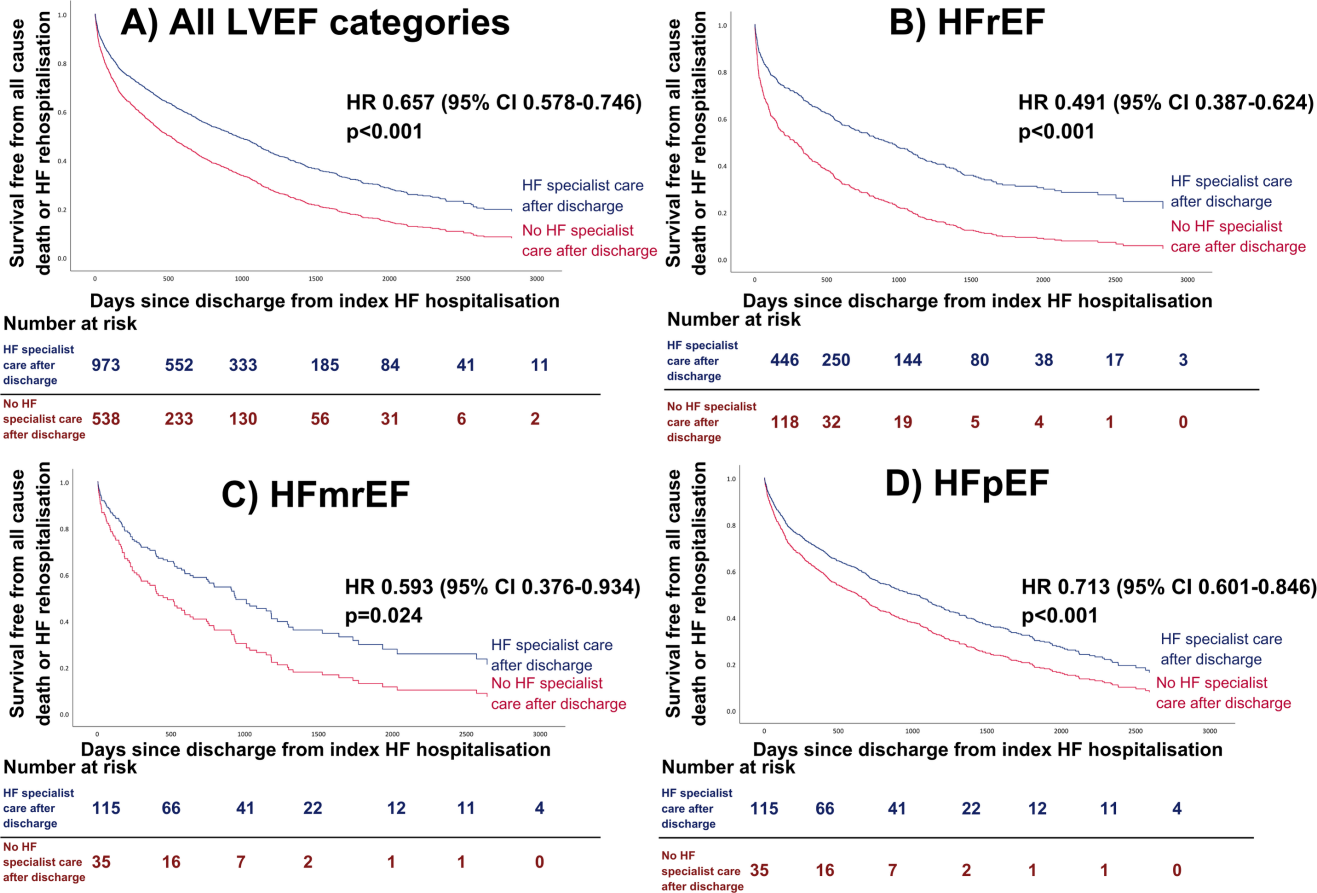
Impact of outpatient HF specialist care following AHF hospitalisation on survival free from a combined endpoint of AHF readmission with AHF or all-cause mortality. (univariable Cox regression analysis). (A) All HF patients. (B) HFrEF. (C) HFmrEF. (D) HFpEF. AHF, acute heart failure; HF, heart failure; HFrEF, HF with reduced EF; HFmrEF, HF with mildly reduced EF; HFpEF, HF with preserved EF; LVEF, left ventricular ejection fraction.

**Figure 2:**
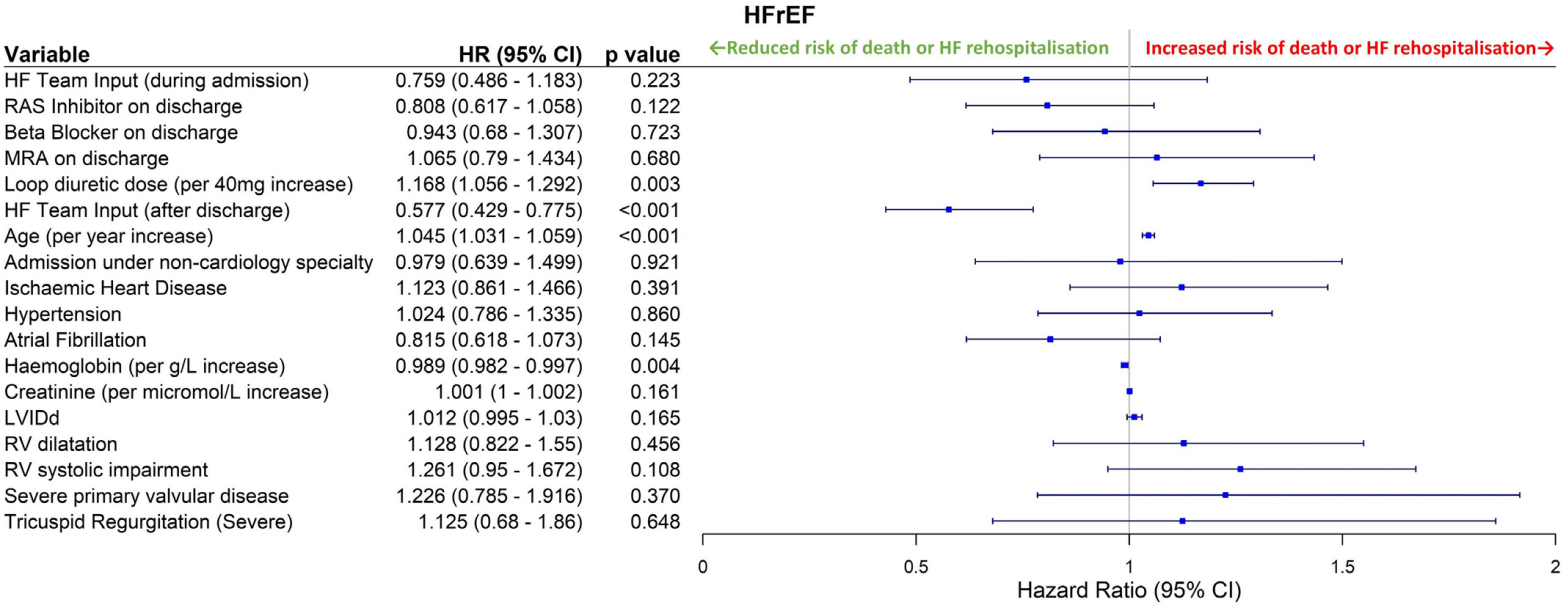
Multivariable Cox regression analysis for HFrEF patients. HRs are for the combined endpoint of all-cause death or heart failure rehospitalisation, with HR>1.0 indicating increased risk. Variables were included in this model if p<0.1 on univariable analysis. EF, ejection fraction; HF, heart failure; HFrEF, HF with reduced EF; MRA, mineralocorticoid receptor antagonist; RAS, renin angiotensin system; LVIDd, Left ventricular internal dimension in diastole, RV, right ventricular.

Cox regression analysis with a combined endpoint of all-cause death or rehospitalisation due to AHF was performed. Postdischarge HF specialist care was associated with significantly improved outcomes on univariable analysis (HR 0.491, 95% CI 0.387 to 0.624, p<0.001, figure 1). On multivariable analysis (figure 2), this effect remained significant (HR 0.577, 0.429–0.775, p<0.001). Conversely, HF specialist care during the index hospitalisation was not associated with significantly improved long-term outcomes after adjusting for covariates (HR 0.759, 0.486–1.183, p=0.223). Higher haemoglobin (HR 0.989 per g/L increase, 0.982–0.997, p=0.004) was independently associated with better outcomes. Older age (HR 1.045 per year of additional age, 1.031–1.059, p<0.001) and higher loop diuretic dose (HR 1.168 per 40 mg furosemide-equivalent increase, 1.056–1.292, p=0.003) were associated with poorer outcomes. Full univariable and multivariable regression analysis results are shown in online supplemental table 3. On the accelerated failure time model, postdischarge HF specialist care (p=0.003) and higher haemoglobin (p=0.007) were associated with increased survival time, while older age (p<0.001) and higher loop diuretic dose (p=0.001) were associated with shorter survival.

#### HF with mildly reduced EF

**Figure 3:**
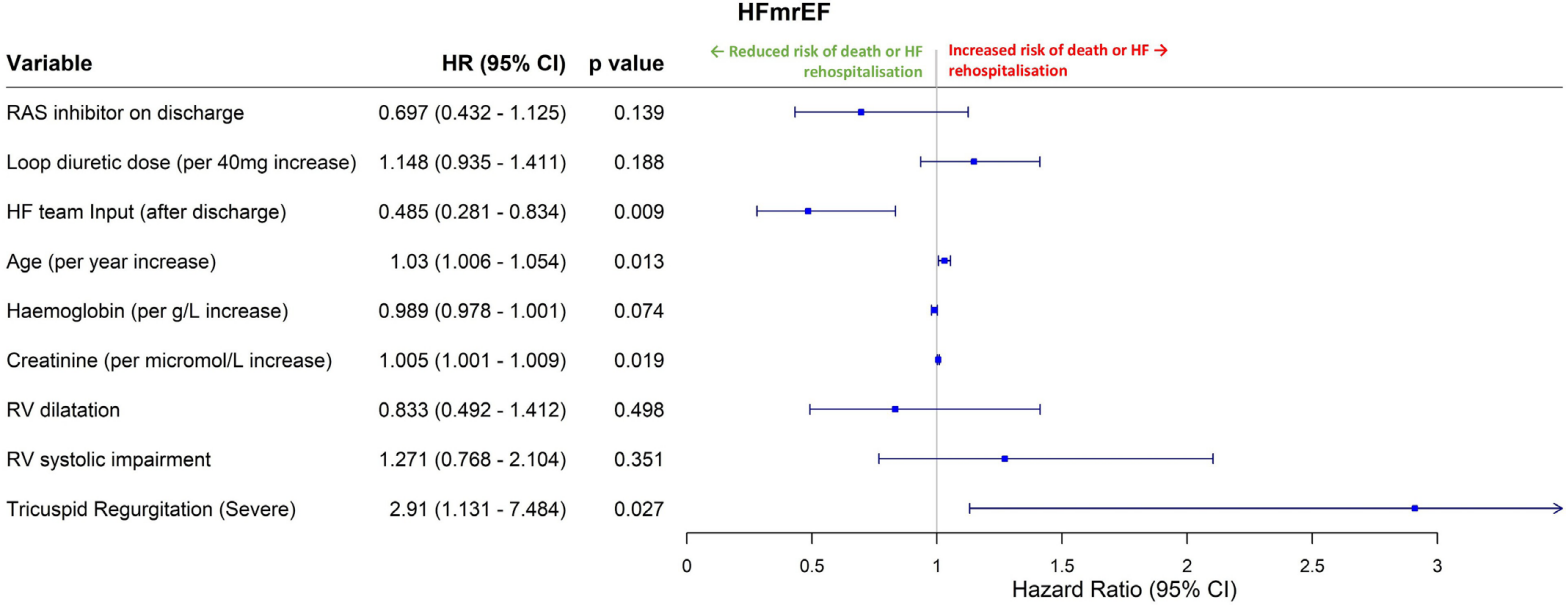
Multivariable Cox regression analysis for HFmrEF patients. HRs are for the combined endpoint of all-cause death or heart failure rehospitalisation, with HR>1.0 indicating increased risk. Variables were included in this model if p<0.1 on univariable analysis. EF, ejection fraction; HF, heart failure; HFmrEF, HF with mildly reduced EF; RAS, renin angiotensin system; RV, right ventricular.

For HFmrEF (figure 3), after adjusting for covariates postdischarge HF specialist care was associated with a significant reduction in the risk of rehospitalisation or death (HR 0.485, 0.281–0.834, p=0.009). Older age (HR 1.030 per additional year of age, 1.006–1.054, p=0.013), higher creatinine (HR 1.005 per micromol/L increase, 1.000–1.009, p=0.019) and severe tricuspid regurgitation (TR) (HR 2.910, 1.131–7.484, p=0.027) were independently associated with worse outcomes. Full univariable and multivariable regression analysis results are shown in online supplemental table 4. On the accelerated failure time model, postdischarge HF specialist care was associated with increased survival (p=0.007), while older age (p=0.003), severe TR (p=0.039) and higher creatinine (p=0.004) were associated with reduced survival duration. Higher loop diuretic dosage was associated with increased risk of adverse events on univariable analysis (HR 1.214 per 40 mg furosemide-equivalent increase, 1.017–1.450, p=0.032), but this association was not significant after adjusting for covariates.

#### HF with preserved EF

**Figure 4:**
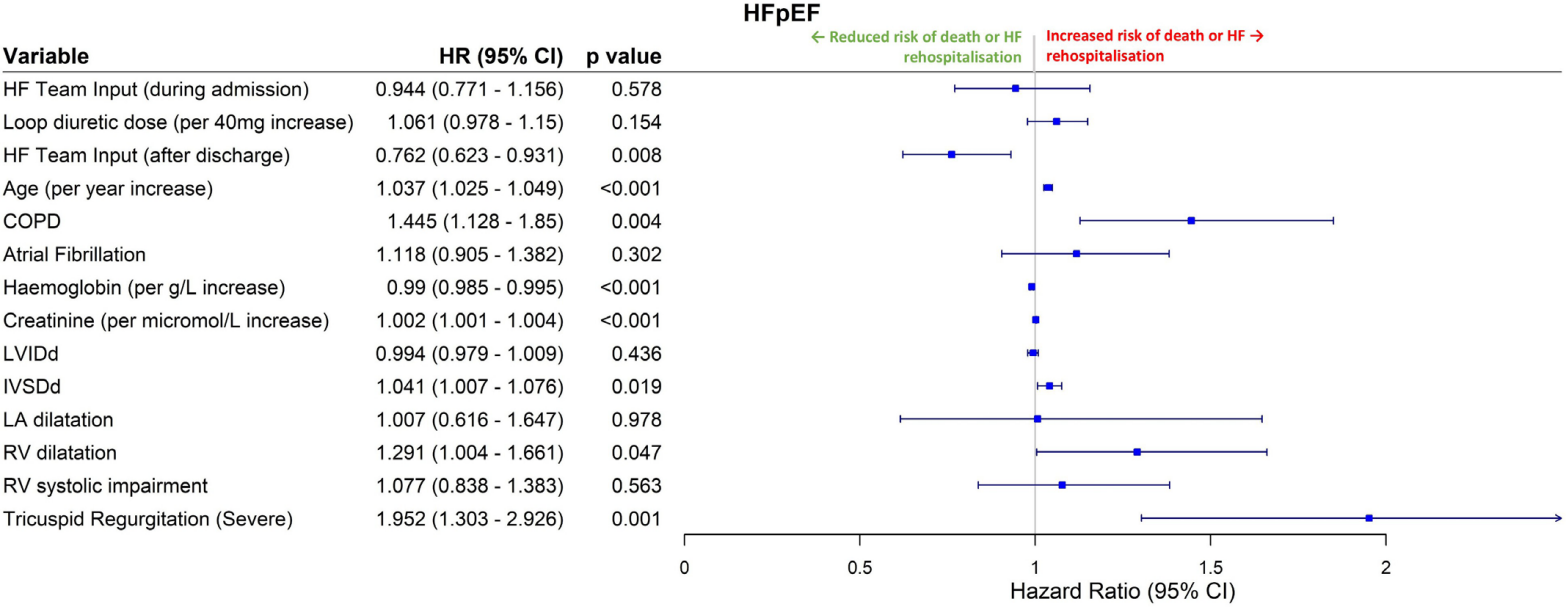
Multivariable Cox regression analysis for HFpEF patients. HRs are for the combined endpoint of all-cause death or heart failure rehospitalisation, with HR>1.0 indicating increased risk. Variables were included in this model if p<0.1 on univariable analysis. COPD, chronic obstructive pulmonary disease; EF, ejection fraction; HF, heart failure; HFpEF, HF with preserved EF; IVSDd, interventricular septal dimension in diastole; LA, left atrium;LVIDd, left ventricular internal dimension in diastole; RV, right ventricular.

For HFpEF, after adjusting for covariates postdischarge HF specialist care was associated with a significant reduction in the risk of rehospitalisation or death (HR 0.762, 0.623–0.931, p=0.008, figure 4), as was higher haemoglobin (HR 0.990 per g/L increase, 0.985–0.995, p<0.001). Older age (HR 1.037 per additional year, 1.025–1.049, p<0.001), coexisting COPD (HR 1.445, 1.128–1.850, p=0.004) and higher creatinine (HR 1.002 per micromol/L increase, 1.001–1.004, p<0.001) were independently associated with poorer long-term survival. On echocardiography, increased interventricular septum diameter (HR 1.041 per mm increase, 1.007–1.076, p=0.019), right ventricular (RV) dilatation (HR 1.291, 1.004–1.661, p=0.047) and severe functional TR (HR 1.952, 1.303–2.926, p=0.001) were independently associated with worse outcomes. Higher loop diuretic dosage was associated with increased risk of adverse events on univariable analysis (HR 1.090 per 40 mg furosemide-equivalent increase, 1.020–1.165, p=0.011), but this association was not significant after adjusting for covariates. Full univariable and multivariable regression analysis results are shown in online supplemental table 5.

## Discussion

In this granular analysis of patients admitted with AHF to hospitals in a single UK county between 2014 and 2022, we found that HF specialist care after discharge was independently associated with substantially reduced risk of rehospitalisation and all-cause mortality over long-term follow-up, irrespective of LVEF, and after adjusting for age, comorbidities and discharge medication regimen. Outpatient HF specialist care was associated with an approximately 50% reduction in the risk of rehospitalisation or death for HFrEF, 40% for HFmrEF and 30% for HFpEF.

The importance of outpatient HF specialist care for HFrEF makes intuitive sense given the opportunity to commence and up-titrate prognostically important GDMT. In our study, patients seen by the HF team after discharge were indeed more likely to end up on ‘three pillars’ of GDMT. However, for HFpEF, a specific role for the HF team has been less clear and hence its association with better long-term outcomes in our study is striking. As well as optimising fluid status, the HF team may improve outcomes in HFpEF by optimising management of ‘upstream’ comorbidities (such as obesity, hypertension and AF) that are increasingly recognised as drivers for the development of HFpEF pathophysiology.^13^ The importance and prevalence of such comorbidities (in our study, 65% of HFpEF patients were hypertensive and 51% had AF) highlights the need for the HF team to include clinicians with expertise in managing a broader range of cardiovascular diseases, rather than becoming excessively subspecialised. This is further emphasised by the fact that almost 1 in 10 patients diagnosed with ‘HF’ with LVEF ≥50% had severe primary valvopathy. A familiarity with such ‘HFpEF mimics’^14^ and their management must be maintained within an expanding HF specialist service. Our findings further highlight the need to review community-based HF service provision in the UK, as 40%–50% of such services currently restrict care to those with low LVEF.^15 16^ Besides being burdensome to patients and associated with poor outcomes,^17^ AHF rehospitalisation represents a significant resource challenge for healthcare systems (the average cost of a ‘second event’ HF hospitalisation in the UK has been estimated at £3461, compared with a ‘first event’ cost of £2444).^18^ Given our findings, commissioning community heart failure services to provide care for all patients with heart failure - regardless of whether they have reduced or preserved LVEF - may prove to be cost-effective by improving patient outcomes. In the future, involvement of the HF specialist team is likely to become ever more important for HFpEF patients as prognostic therapies beyond SGLT2 inhibitors emerge from clinical trials into practice guidelines^19^ and the focus of treatment during and after AHF hospitalisation extends beyond decongestion. Our finding that increased interventricular septal diameter, RV dilatation and severe TR are all independently associated with worse outcomes in HFpEF aligns with current understanding of HFpEF pathophysiology, as increased filling pressures drive LV remodelling and cause back-pressure on the RV. These features could potentially contribute to future prognostic tools for the echocardiographic assessment of HFpEF patients.

Long-term survival and risk of readmission to hospital due to AHF did not differ significantly across the LVEF spectrum. However, while the majority of deaths in the HFrEF cohort were due to cardiovascular causes (55%), this was not the case in HFmrEF (45%) or HFpEF (39%). Taken together with the finding that HFpEF patients were on average older and with more non-cardiovascular comorbidities such as COPD, an impression emerges that the relative significance of HF for patient outcomes may vary across the LVEF spectrum. This has been suggested by previous analyses demonstrating that a patient’s comorbidity burden is more predictive of prognosis in HFpEF than in HFrEF.^20^ Holistic medical assessment and an effective interface between the HF team and other medical specialties may, therefore, be expected to achieve further improvements in HFpEF patient experience and outcomes.

This analysis also identifies opportunities for improvement in HFrEF care. HFrEF patients discharged from their index hospitalisation on suboptimal medical therapy generally remained on suboptimal medical therapy: 67% saw no change or a decrease in the number of GDMT medications prescribed between index discharge and final encounter. Overall, rates of HFrEF GDMT prescription were suboptimal in the cohort we studied, but appear consistent with registry data suggesting prescription rates for all three of RAS inhibitor, beta blocker and MRA are as low as 25%–30%^21^ in some HF cohorts. The STRONG-HF trial supports the rapid initiation and up-titration of GDMT in the first 2 weeks after hospitalisation for AHF,^22^ and as such a patient’s time in hospital should be seen as a key opportunity to optimise treatment at the frontend of their HF journey. In our study, HFrEF rehospitalisation due to AHF occurred at a median of 74 days following discharge, further highlighting that ‘upfront’ medication optimisation and early HF team input is essential to prevent adverse outcomes. Our finding that higher loop diuretic doses are associated with poorer outcomes (unadjusted relative risk increase per 40 mg furosemide-equivalent dose increase of 23% in HFrEF, 21% in HFmrEF and 9% in HFpEF) is consistent with observational data reported elsewhere for these cohorts.^23 24^ This supports the use of loop diuretic dosage as an enrichment factor for future clinical trials in HF across the LVEF spectrum.

Finally, in this study, 38% of patients did not attend the first scheduled outpatient contact with the specialist HF team. This non-attendance figure is significantly higher than the UK average for outpatient appointments (~23%^25^ and further investigation is warranted to understand whether there are specific challenges in arranging outpatient care for the HF cohort that need to be addressed). Non-attendees in our study represented a particularly elderly segment of the cohort, perhaps suggesting the presence of additional technological and logistical barriers that may disproportionately prevent older individuals from accessing outpatient HF services. These could include difficulties travelling to outpatient departments for less mobile patients, potentially building a case for greater delivery of HF care within the community through outreach and ‘hospital at home’ services.

### Limitations

As with all retrospective analyses, our findings are associative rather than providing evidence of causality and are vulnerable to confounding. However, given the strong independent association of HF specialist care with a significant improvement in clinical outcomes in multiple models, we are confident of our broad conclusions.

Our study period ended in 2022 and so does not cover the era of SGLT2 inhibitors for HFpEF and overlaps minimally with their guideline-directed use for HFrEF, potentially limiting generalisability of our results to contemporary practice. Care should also be taken when generalising results from this study of a single county in the UK that comprises a relatively elderly, overwhelmingly Caucasian population. HFmrEF represents a relatively poorly studied subgroup, as historically many observational studies and clinical trials have dichotomised the HF cohort at LVEF=40%. Where specific data are available for HFmrEF, these patients typically constitute only 10%–19% of HF cohorts.^26 27^ In line with this, HFmrEF accounted for approximately 10% of the cohort in our survival analysis and the relatively small number of patients in this group should be considered when interpreting our results.

Definition of LVEF is challenging in longitudinal studies of HF patients. In our study, we have used the LVEF at/closest to the time of AHF hospitalisation to define our subgroups to ensure a consistent approach to data handling. Measurement of LVEF in the context of AHF may lead to overestimation or underestimation of systolic function due to abnormal loading conditions among other factors, and there is no doubt a single snapshot LVEF represents an oversimplification of a patient’s systolic function phenotype in any case. However, as we found that all LVEF groups benefited from postdischarge HF specialist care, this uncertainty should not detract from the key messages of our study.

## Conclusion

Outpatient HF specialist care following hospitalisation for AHF is independently associated with improved long-term outcomes, irrespective of LVEF. All patients hospitalised with AHF should, therefore, be considered for outpatient HF team care, irrespective of LVEF category and comorbidity profile.

## Supplementary material

10.1136/openhrt-2025-003432 online supplemental file 1

## Data Availability

Data are available upon reasonable request.
