# Supplementary material for: Outpatient heart failure specialist care following acute heart failure hospitalisation improves long-term outcomes
Source: Open Heart. 2025 Sep 29;12(2):e003432. doi: 10.1136/openhrt-2025-003432 (PMC12481318; doi:10.1136/openhrt-2025-003432)
Supplement: online supplemental file 1 [file openhrt-12-2-s001.docx]

Supplemental Table 1: Echocardiogram findings for the studied cohort. Echocardiogram data were available for 1743 patients. Where multiple echocardiogram studies were available for a single patient, the examination performed closest to the date of index heart failure hospitalisation was selected.

|  | Total Cohort (n=1743) | LVEF ≤40% (n=622) | LVEF 41-49% (n=165) | LVEF≥50% (n=956) | p value |
| --- | --- | --- | --- | --- | --- |
| LV Ejection Fraction, median (IQR)  [count of available data] | 53% (34-63)  [n=1743] | 30% (23-35)  [n=622] | 45% (43-48)  [n=165] | 63% (58-68)  [n=956] |  |
| **LV Internal Diameter in Diastole (mm), median (IQR)** | **50 (44-57)**  [n=1668] | **58 (52-63)^1,2^**  [n=594] | **51 (45-56)^3^**  [n=153] | **46 (41-50)**  [n=921] | **<0.001^b^** |
| **Interventricular Septum in Diastole (mm), median (IQR)** | **12 (10-13)**  [n=1647] | **11 (10-12)^1,2^**  [n=582] | **12 (11-14)**  [n=149] | **12 (11-14)**  [n=916] | **<0.001^b^** |
| **E/A Ratio, median (IQR)** | **0.96 (0.72-1.54)**  [n=687] | **1.36 (0.81-1.98)^1,2^**  [n=220] | **0.83 (0.69-1.37)**  [n=57] | **0.88 (0.68-1.33)**  [n=410] | **<0.001^b^** |
| **E/E’ Ratio, median (IQR)** | **13.5 (10.4-18.6)**  [n=469] | **15.4 (11.3-21.0)^1,2^**  [n=168] | **12.3 (9.7-17.0)**  [n=47] | **12.5 (9.7-17.8)**  [n=254] | **<0.001^b^** |
| **Left Atrial Dilatation, n (%)** | **1615 (93%)**  [n=1719] | **586 (94%)^2^**  [n=604] | **150 (91%)**  [n=162] | **879 (92%)**  [n=953] | **0.010^a^** |
| Right Ventricular Dilatation, n (%) | 617 (35%)  [n=1697] | 230 (37%)  [n=594] | 51 (31%)  [n=160] | 336 (35%)  [n=943] | 0.322^a^ |
| **Right Ventricular Systolic Dysfunction, n (%)** | **642 (37%)**  [n=1685] | **327 (53%)^1,2^**  [n=589] | **51 (31%)**  [n=159] | **264 (28%)**  [n=937] | **<0.001^a^** |
| **Right Ventricular Systolic Pressure (mmHg), median (IQR)** | **48 (39-59)**  [n=1203] | **48 (40-56)**  [n=427] | **46 (35-57)^3^**  [n=106] | **48 (40-62)**  [n=670] | **0.038^b^** |
| High Echocardiographic Probability of Pulmonary Hypertension, n (%) | 18 (1%)  [n=1610] | 7 (1%)  [n=561] | 2 (1%)  [n=156] | 9 (1%)  [n=893] | 0.144^c^ |
| **Valvular Heart Disease** | | | | | |
| Aortic Stenosis | [n=1718] | [n=601] | [n=163] | [n=954] |  |
| **Mild** | **193 (11%)** | **51 (8%)^2^** | **17 (10%)** | **125 (13%)** | **0.010^a^** |
| Moderate | 127 (7%) | 41 (7%) | 13 (8%) | 73 (8%) | 0.704^a^ |
| Severe | 95 (6%) | 39 (6%) | 7 (4%) | 49 (5%) | 0.479^a^ |
| Aortic Regurgitation | [n=1717] | [n=600] | [n=163] | [n=954] |  |
| Mild | 507 (29%) | 172 (28%) | 46 (28%) | 289 (30%) | 0.511^a^ |
| Moderate | 83 (5%) | 37 (6%) | 6 (4%) | 40 (4%) | 0.213^a^ |
| Severe | 9 (1%) | 4 (1%) | 0 (0%) | 5 (1%) | 0.952^c^ |
| Mitral Stenosis | [n=1713] | [n=601] | [n=162] | [n=950] |  |
| **Mild** | **45 (3%)** | **8 (1%)^2^** | **4 (2%)** | **33 (3%)** | **0.030^c^** |
| Moderate | 13 (0.7%) | 1 (0.2%)^1,2^ | 1 (1%) | 11 (1%) | 0.081^c^ |
| Severe | 3 (0.2%) | 1 (0.2%) | 0 (0%) | 2 (0.2%) | 0.561^c^ |
| Mitral Regurgitation (Primary) | [n=1728] | [n=606] | [n=163] | [n=959] |  |
| **Mild** | **510 (30%)** | **88 (14%)^1,2^** | **59 (36%)** | **363 (38%)** | **<0.001^a^** |
| **Moderate** | **143 (8%)** | **30 (5%)^1,2^** | **15 (9%)** | **98 (10%)** | **<0.001^a^** |
| **Severe** | **30 (2%)** | **5 (0.8%)^2^** | **2 (1%)** | **23 (2%)** | **0.050^c^** |
| Mitral Regurgitation (Functional) | [n=1728] | [n=606] | [n=163] | [n=959] |  |
| **Mild** | **393 (23%)** | **217 (35%)^1,2^** | **39 (24%)^3^** | **137 (14%)** | **<0.001^a^** |
| **Moderate** | **229 (13%)** | **169 (27%)^1,2^** | **9 (5%)** | **51 (5%)** | **<0.001^a^** |
| **Severe** | **45 (3%)** | **35 (5%)^1,2^** | **3 (2%)** | **7 (1%)** | **<0.001^a^** |
| Tricuspid Regurgitation | [n=1721] | [n=603] | [n=163] | [n=955] |  |
| Mild | 721 (41%) | 267 (43%) | 65 (39%) | 389 (41%) | 0.586^a^ |
| Moderate | 326 (19%) | 127 (20%) | 25 (15%) | 174 (18%) | 0.255^a^ |
| Severe | 177 (10%) | 57 (9%) | 12 (7%) | 108 (11%) | 0.170^a^ |
|  | | | | | |
| a – Chi Square test; b – Independent Samples Kruskal Wallis one way ANOVA; c – Fisher exact test  Each superscript number denotes a significant difference between columns on post-hoc pairwise comparison (p<0.05 with Bonferroni correction): 1 - LVEF ≤40% vs LVEF 41-49% 2 - LVEF ≤40% vs LVEF≥50% 3 - LVEF 41-49% vs LVEF≥50%. | | | | | |

Supplemental Table 2: Variables assessed on univariable Cox regression analysis (composite end point of all cause death or rehospitalisation due to heart failure). Variables with p<0.1 on univariable analysis were included in a multivariable model, the results of which are reported in the main figures.

| **Demographics** |
| --- |
| Age |
| Male sex |
| Non-caucasian ethnicity |
| **Past Medical History** |
| Ischaemic heart disease |
| Hypertension |
| Diabetes mellitus |
| Chronic obstructive pulmonary disease |
| Atrial Fibrillation |
| **Blood Results on Index Heart Failure Admission** |
| Haemoglobin |
| Creatinine |
| Sodium |
| Potassium |
| **Echocardiography** |
| Left ventricular internal diameter at end diastole |
| Interventricular septal diameter at end diastole |
| Left atrial dilatation |
| Right ventricular dilatation |
| Right ventricular systolic impairment |
| Severe primary valvular disease |
| Severe tricuspid regurgitation |
| **Medications prescribed on discharge** |
| Renin-angiotensin system inhibitor |
| Beta Blocker |
| Mineralocorticoid receptor antagonist |
| Loop diuretic dose |
| **HF Team Input** |
| Index admission under non-cardiology specialty |
| Inpatient Heart Failure Team Input |
| Outpatient Heart Failure Team Input post-discharge |


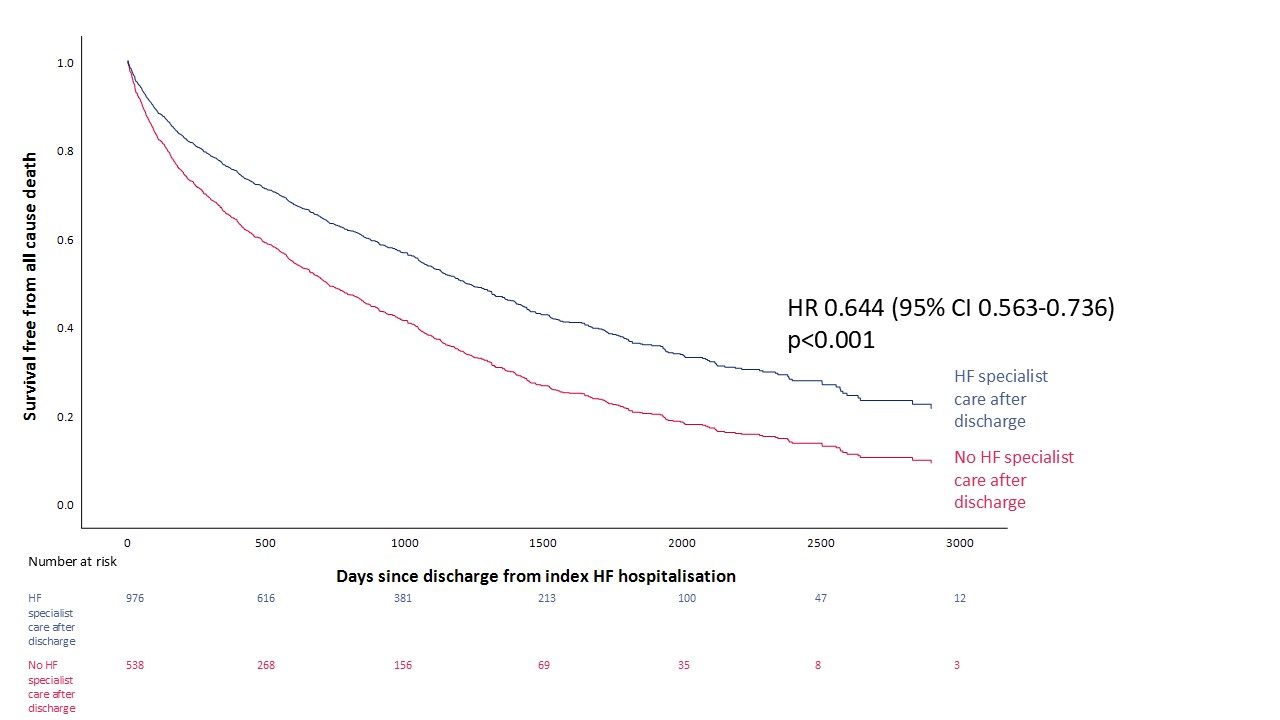


Supplemental Figure 1: Survival curves from univariable Cox regression analysis comparing all-cause mortality for those who received HF specialist care after discharge versus those who did not across all LVEF categories.

*
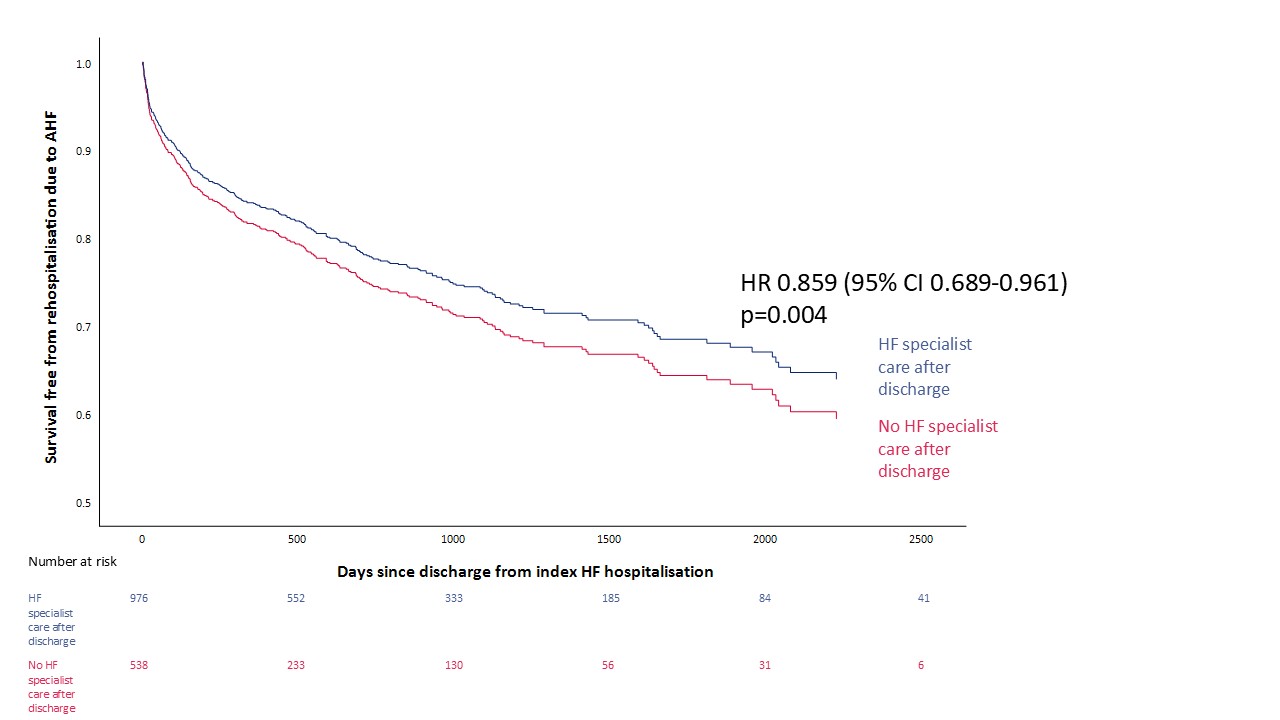
*

Supplemental Figure 2: Survival curves from univariable Cox regression analysis comparing survival free from rehospitalisation due to AHF for those who received HF specialist care after discharge versus those who did not across all LVEF categories.

Supplemental Table 3: Univariable and multivariable Cox regression results for HFrEF (LVEF≤40%). Hazard ratios (HR) represent the risk of the combined endpoint of all-cause death or rehospitalisation due to heart failure. HR values greater than 1 indicate an increased risk of this adverse outcome, while HR values less than 1 indicate reduced risk of adverse outcomes.

| Variable for patients with HFrEF | Univariable Analysis | | | Multivariable Analysis | | |
| --- | --- | --- | --- | --- | --- | --- |
|  | HR | (95% CI) | p value | HR | (95% CI) | p value |
| **Age (per year increase)** | **1.051** | **(1.040 – 1.061)** | **<0.001** | **1.045** | **(1.031 – 1.059)** | **<0.001** |
| Male sex | 0.913 | (0.729 – 1.143) | 0.426 |  |  |  |
| Non-Caucasian ethnicity | 0.902 | (0.517 – 1.575) | 0.717 |  |  |  |
| **Non-cardiology admission** | **1.614** | **(1.305 – 1.995)** | **<0.001** | 0.979 | (0.639 – 1.499) | 0.921 |
| **Ischaemic Heart Disease** | **1.641** | **(1.321 – 2.038)** | **<0.001** | 1.123 | (0.861 – 1.466) | 0.391 |
| **Hypertension** | **1.299** | **(1.044 – 1.617)** | **0.019** | 1.024 | (0.786 – 1.335) | 0.860 |
| Diabetes Mellitus | 1.197 | (0.961 – 1.490) | 0.109 |  |  |  |
| COPD | 1.242 | (0.908 – 1.701) | 0.176 |  |  |  |
| **Atrial Fibrillation** | **1.316** | **(1.069 – 1.620)** | **0.010** | 0.815 | (0.618 – 1.073) | 0.145 |
| **Blood Results** | | | | | | |
| **Haemoglobin (per g/L increase)** | **0.979** | **(0.973 – 0.984)** | **<0.001** | **0.989** | **(0.982 – 0.997)** | **0.004** |
| **Creatinine (per micromol/L increase)** | **1.002** | **(1.001 - 1.002)** | **<0.001** | 1.001 | (1.000 – 1.002) | 0.161 |
| Sodium | 0.984 | (0.963 – 1.006) | 0.146 |  |  |  |
| Potassium | 0.872 | (0.713 – 1.006) | 0.181 |  |  |  |
| **Echocardiography** | | | | | | |
| **LVIDd** | **0.987** | **(0.975 – 0.999)** | **0.032** | 1.012 | (0.995 – 1.030) | 0.165 |
| IVSDd | 1.032 | (0.987 – 1.079) | 0.172 |  |  |  |
| LA dilatation | 1.617 | (0.765 – 3.418) | 0.208 |  |  |  |
| **RV dilatation** | **1.364** | **(1.099 – 1.694)** | **0.005** | 1.128 | (0.822 – 1.550) | 0.456 |
| **RV systolic impairment** | **1.246** | **(1.003 – 1.546)** | **0.047** | 1.261 | (0.950 – 1.672) | 0.108 |
| Severe primary valvular disease | 1.398 | (0.972 – 2.012) | 0.071 | 1.226 | (0.785 – 1.916) | 0.370 |
| Mild TR | 1.169 | (0.888 – 1.538) | 0.266 |  |  |  |
| **Moderate TR** | **1.511** | **(1.098 – 2.079)** | **0.011** | 1.008 | (0.667 – 1.523) | 0.971 |
| **Severe TR** | **2.523** | **(1.728 – 3.684)** | **<0.001** | 1.125 | (0.680 – 1.860) | 0.648 |
| **Medications** | | | | | | |
| **RAS Inhibitor** | **0.532** | **(0.431 – 0.657)** | **<0.001** | 0.808 | (0.617– 1.058) | 0.122 |
| **Beta Blocker** | **0.713** | **(0.551 – 0.923)** | **0.010** | 0.943 | (0.680 – 1.307) | 0.723 |
| **MRA** | **0.615** | **(0.498 – 0.759)** | **<0.001** | 1.065 | (0.790 – 1.434) | 0.680 |
| **Loop Diuretic Dose (per 40mg Furosemide-equivalent increase)** | **1.231** | **(1.140 – 1.329)** | **<0.001** | **1.168** | **(1.056 – 1.292)** | **0.003** |
| **HF Team Input** | | | | | | |
| **Inpatient** | **0.626** | **(0.499 – 0.786)** | **<0.001** | 0.759 | (0.486 – 1.183) | 0.223 |
| **Outpatient** | **0.491** | **(0.387 – 0.624)** | **<0.001** | **0.577** | **(0.429 – 0.775)** | **<0.001** |
|  | | | | | | |
| **Abbreviations**  AF - Atrial Fibrillation; COPD – Chronic Obstructive Pulmonary Disease; CVA – Cerebrovascular Accident; eGFR – estimated Glomerular Filtration Rate; IVSDd – Interventricular Septal Diameter in Diastole; LA – Left Atrium; LVEF – Left Ventricular Ejection Fraction; LVIDd – Left Ventricular Internal Diameter in Diastole; MRA – Mineralocorticoid Receptor Antagonist; NT-proBNP - N-Terminal prohormone of Brain Natriuretic Peptide; RAS - Renin-Angiotensin Aldosterone System; RV – Right Ventricle; SGLT2i – Sodium-Glucose Co-transporter 2 Inhibitor; SPAP - Systolic Pulmonary Arterial Pressure; TR – Tricuspid Regurgitation. | | | | | | |

Supplemental Table 4: Univariable and multivariable Cox regression results for HFmrEF (LVEF 41-49%). Hazard ratios (HR) represent the risk of the combined endpoint of all-cause death or rehospitalisation due to heart failure. HR values greater than 1 indicate an increased risk of this adverse outcome, while HR values less than 1 indicate reduced risk of adverse outcomes.

| Variables for patients with HFmrEF | Univariable Analysis | | | Multivariable Analysis | | |
| --- | --- | --- | --- | --- | --- | --- |
|  | HR | (95% CI) | p value | HR | (95% CI) | p value |
| **Age (per year increase)** | **1.049** | **(1.028 – 1.070)** | **<0.001** | **1.030** | **(1.006 – 1.054)** | **0.013** |
| Male gender | 1.181 | (0.789 – 1.769) | 0.419 |  |  |  |
| Non-Caucasian ethnicity | 0.841 | (0.206 – 3.439) | 0.810 |  |  |  |
| Non-cardiology admission | 1.227 | (0.820 – 1.837) | 0.321 |  |  |  |
| Ischaemic Heart Disease | 1.295 | (0.859 – 1.952) | 0.217 |  |  |  |
| Hypertension | 1.227 | (0.793 – 1.898) | 0.358 |  |  |  |
| Diabetes Mellitus | 0.857 | (0.556 – 1.319) | 0.482 |  |  |  |
| COPD | 1.351 | (0.787 – 2.318) | 0.276 |  |  |  |
| Atrial Fibrillation | 1.217 | (0.814 – 1.820) | 0.339 |  |  |  |
| **Blood results** | | | | | | |
| **Haemoglobin (per g/L increase)** | **0.979** | **(0.968 – 0.990)** | **<0.001** | 0.989 | (0.978 – 1.001) | 0.074 |
| **Creatinine (per micromol/L increase)** | **1.006** | **(1.003 – 1.010)** | **<0.001** | **1.005** | **(1.001 – 1.009)** | **0.019** |
| Sodium | 0.971 | (0.924 – 1.020) | 0.244 |  |  |  |
| Potassium | 1.261 | (0.842 – 1.888) | 0.261 |  |  |  |
| **Echocardiography** | | | | | | |
| LVIDd | 0.975 | (0.946 – 1.005) | 0.101 |  |  |  |
| IVSDd | 0.998 | (0.938 – 1.062) | 0.949 |  |  |  |
| LA dilatation | 1.618 | (0.656 – 3.993) | 0.296 |  |  |  |
| **RV dilatation** | **1.458** | **(0.952 – 2.233)** | **0.083** | 0.833 | (0.492 – 1.412) | 0.498 |
| **RV systolic impairment** | **1.685** | **(1.106 – 2.567)** | **0.015** | 1.271 | (0.768 – 2.104) | 0.351 |
| Severe primary valvular heart disease | 1.035 | (0.379 – 2.824) | 0.947 |  |  |  |
| **Mild TR** | **1.813** | **(1.122 – 2.930)** | **0.015** | 1.558 | (0.860 – 2.823) | 0.144 |
| **Moderate TR** | **2.778** | **(1.499 – 5.147)** | **0.001** | **2.121** | **(1.008 – 4.460)** | **0.047** |
| **Severe TR** | **3.458** | **(1.478 – 8.093)** | **0.004** | **2.910** | **(1.131 – 7.484)** | **0.027** |
| **Medications** | | | | | | |
| **RAS inhibitor** | **0.367** | **(0.244 – 0.554)** | **<0.001** | 0.697 | (0.432 – 1.125) | 0.139 |
| Beta Blocker | 1.023 | (0.640 – 1.636) | 0.923 |  |  |  |
| MRA | 1.013 | (0.670 – 1.531) | 0.951 |  |  |  |
| **Loop diuretic dose (per 40mg Furosemide-equivalent increase)** | **1.214** | **(1.017 – 1.450)** | **0.032** | 1.148 | (0.935 – 1.411) | 0.188 |
| **HF team Input** | | | | | | |
| Inpatient | 0.831 | (0.625 – 1.459) | 0.831 |  |  |  |
| **Outpatient** | **0.593** | **(0.376 – 0.934)** | **0.024** | **0.485** | **(0.281 – 0.834)** | **0.009** |
|  | | | | | | |
| **Abbreviations**  AF - Atrial Fibrillation; COPD – Chronic Obstructive Pulmonary Disease; CVA – Cerebrovascular Accident; eGFR – estimated Glomerular Filtration Rate; IVSDd – Interventricular Septal Diameter in Diastole; LA – Left Atrium; LVEF – Left Ventricular Ejection Fraction; LVIDd – Left Ventricular Internal Diameter in Diastole; MRA – Mineralocorticoid Receptor Antagonist; NT-proBNP - N-Terminal prohormone of Brain Natriuretic Peptide; RAS – Renin-Angiotensin Aldosterone System; RV – Right Ventricle; SGLT2i – Sodium-Glucose Co-transporter 2 Inhibitor; SPAP - Systolic Pulmonary Arterial Pressure; TR – Tricuspid Regurgitation. | | | | | | |

Supplemental Table 5: Univariable and multivariable Cox regression results for HFpEF (LVEF ≥50%). Hazard ratios (HR) represent the risk of the combined endpoint of all-cause death or rehospitalisation due to heart failure. HR values greater than 1 indicate an increased risk of this adverse outcome, while HR values less than 1 indicate reduced risk of adverse outcomes.

| Variables for patients with HFpEF | Univariable Analysis | | | Multivariable Analysis | | |
| --- | --- | --- | --- | --- | --- | --- |
|  | HR | (95% CI) | p value | HR | (95% CI) | p value |
| **Age (per year increase)** | **1.041** | **(1.032 - 1.050)** | **<0.001** | **1.037** | **(1.025 – 1.049)** | **<0.001** |
| Male gender | 0.997 | (0.841 - 1.182) | 0.972 |  |  |  |
| Non-Caucasian ethnicity | 1.001 | (0.686 – 1.460) | 0.927 |  |  |  |
| Non-cardiology admission | 1.186 | (0.997 – 1.411) | 0.053 |  |  |  |
| Ischaemic Heart Disease | 0.995 | (0.832 – 1.190) | 0.958 |  |  |  |
| Hypertension | 1.068 | (0.887 – 1.285) | 0.489 |  |  |  |
| Diabetes Mellitus | 1.006 | (0.843 – 1.201) | 0.945 |  |  |  |
| **COPD** | **1.245** | **(0.999 – 1.550)** | **0.051** | **1.445** | **(1.128 – 1.850)** | **0.004** |
| **Atrial Fibrillation** | **1.343** | **(1.132 – 1.593)** | **<0.001** | 1.118 | (0.905 – 1.382) | 0.302 |
| **Blood results** | | | | | | |
| **Haemoglobin (per g/L increase)** | **0.983** | **(0.979 – 0.988)** | **<0.001** | **0.990** | **(0.985 – 0.995)** | **<0.001** |
| **Creatinine (per micromol/L increase)** | **1.003** | **(1.001 – 1.004)** | **<0.001** | **1.002** | **(1.001 – 1.004)** | **<0.001** |
| Sodium | 1.008 | (0.988 – 1.028) | 0.454 |  |  |  |
| Potassium | 1.020 | (0.876 – 1.186) | 0.801 |  |  |  |
| **Echocardiography** | | | | | | |
| **LVIDd (per mm increase)** | **0.986** | **(0.973 – 0.998)** | **0.027** | 0.994 | (0.979 – 1.009) | 0.436 |
| **IVSDd (per mm increase)** | **1.030** | **(0.996 – 1.065)** | **0.087** | **1.041** | **(1.007 – 1.076)** | **0.019** |
| **LA dilatation** | **1.547** | **(1.027 – 2.331)** | **0.037** | 1.007 | (0.616 – 1.647) | 0.978 |
| **RV dilatation** | **1.781** | **(1.491 – 2.127)** | **<0.001** | **1.291** | **(1.004 – 1.661)** | **0.047** |
| **RV systolic impairment** | **1.692** | **(1.403 – 2.040)** | **<0.001** | 1.077 | (0.838 – 1.383) | 0.563 |
| **Mild TR** | **1.358** | **(1.095 – 1.684)** | **0.005** | 0.995 | (0.765 – 1.292) | 0.968 |
| **Moderate TR** | **2.013** | **(1.561 – 2.598)** | **<0.001** | 1.292 | (0.924 – 1.805) | 0.134 |
| **Severe TR** | **3.558** | **(2.655 – 4.770)** | **<0.001** | **1.952** | **(1.303 – 2.926)** | **0.001** |
| **Medications** | | | | | | |
| RAS Inhibitor | 0.884 | (0.738 – 1.058) | 0.178 |  |  |  |
| Beta Blocker | 0.883 | (0.739 – 1.055) | 0.171 |  |  |  |
| MRA | 1.097 | (0.902 – 1.333) | 0.355 |  |  |  |
| **Loop diuretic dose (per 40mg Furosemide-equivalent increase)** | **1.090** | **(1.020 – 1.165)** | **0.011** | 1.061 | (0.978 – 1.150) | 0.154 |
| **HF Team Input** | | | | | | |
| **Inpatient** | **0.836** | **(0.705 – 0.992)** | **0.040** | 0.944 | (0.771 – 1.156) | 0.578 |
| **Outpatient** | **0.713** | **(0.601 – 0.846)** | **<0.001** | **0.762** | **(0.623 – 0.931)** | **0.008** |
|  | | | | | | |
| **Abbreviations**  AF - Atrial Fibrillation; COPD – Chronic Obstructive Pulmonary Disease; CVA – Cerebrovascular Accident; eGFR – estimated Glomerular Filtration Rate; IVSDd – Interventricular Septal Diameter in Diastole; LA – Left Atrium; LVEF – Left Ventricular Ejection Fraction; LVIDd – Left Ventricular Internal Diameter in Diastole; MRA – Mineralocorticoid Receptor Antagonist; NT-proBNP - N-Terminal prohormone of Brain Natriuretic Peptide; RAS - RAS – Renin-Angiotensin Aldosterone System; RV – Right Ventricle; SGLT2i – Sodium-Glucose Co-transporter 2 Inhibitor; SPAP - Systolic Pulmonary Arterial Pressure; TR – Tricuspid Regurgitation | | | | | | |
